# Supplementary figures and images for: Prevalence and type distribution of human papillomavirus in a Chinese urban population between 2014 and 2018: a retrospective study
Source: PeerJ. 2020 Mar 23;8:e8709. doi: 10.7717/peerj.8709 (PMC7098390; doi:10.7717/peerj.8709)

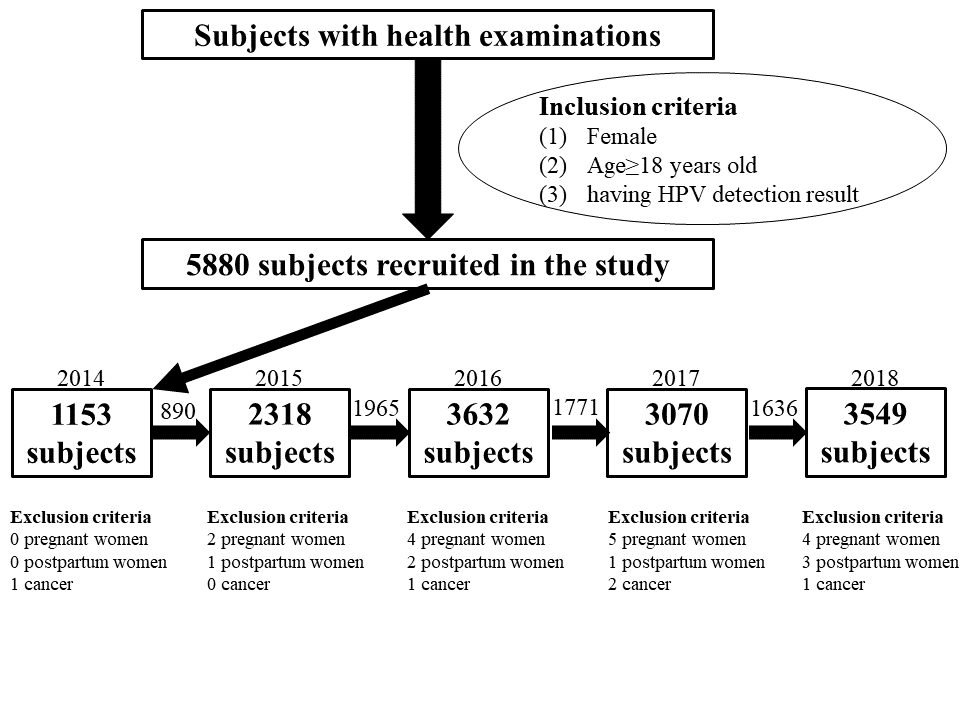

Supplement: Figure S1 [file peerj-08-8709-s001.png]

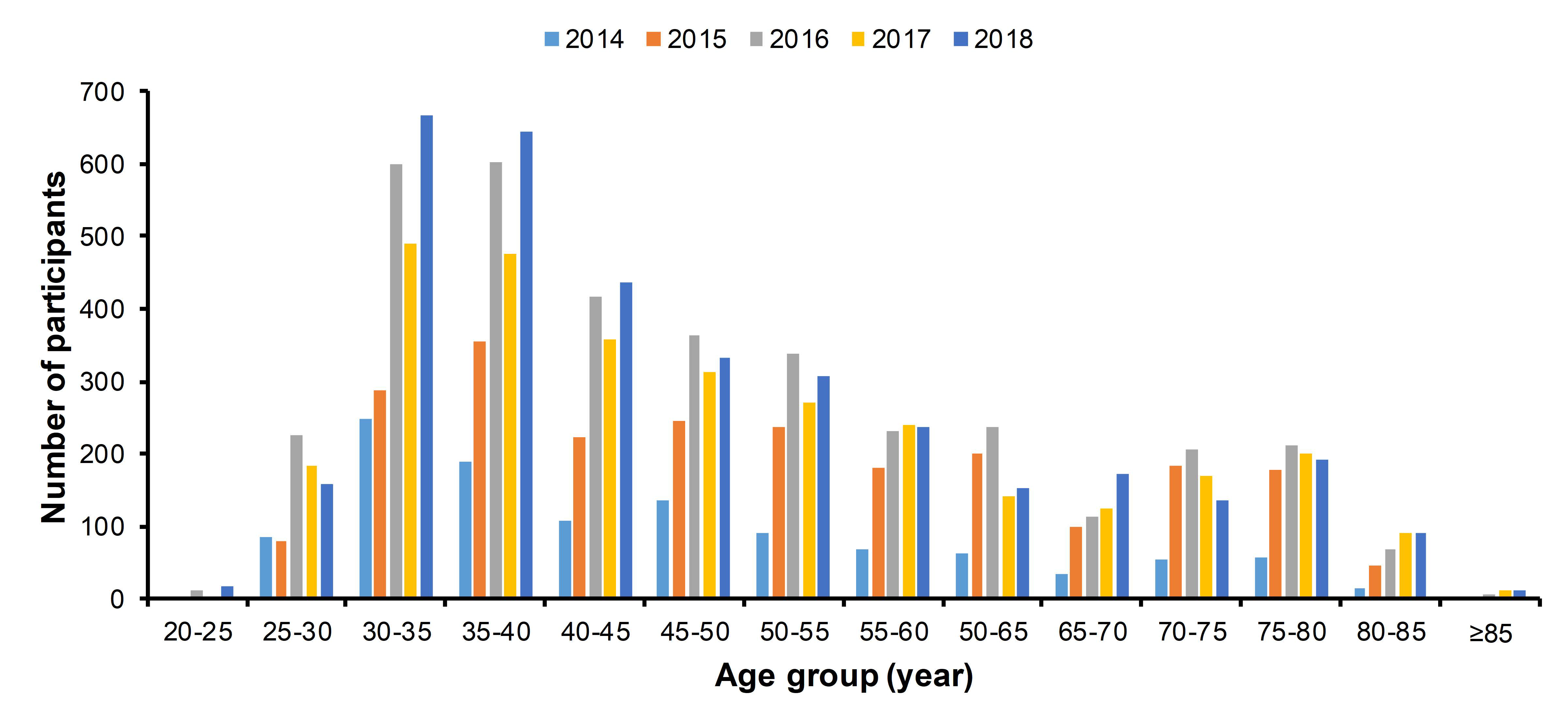

Supplement: Figure S2 — (A) prevalence rate of low risk and high risk HPV types in each year; (B) prevalence of all HPV types of different age groups; (C) prevalence of low risk HPV types of different age groups; (D) prevalence of high risk HPV types of different age groups; (E) prevalence of HPV06 and HPV11. [file peerj-08-8709-s002.png]

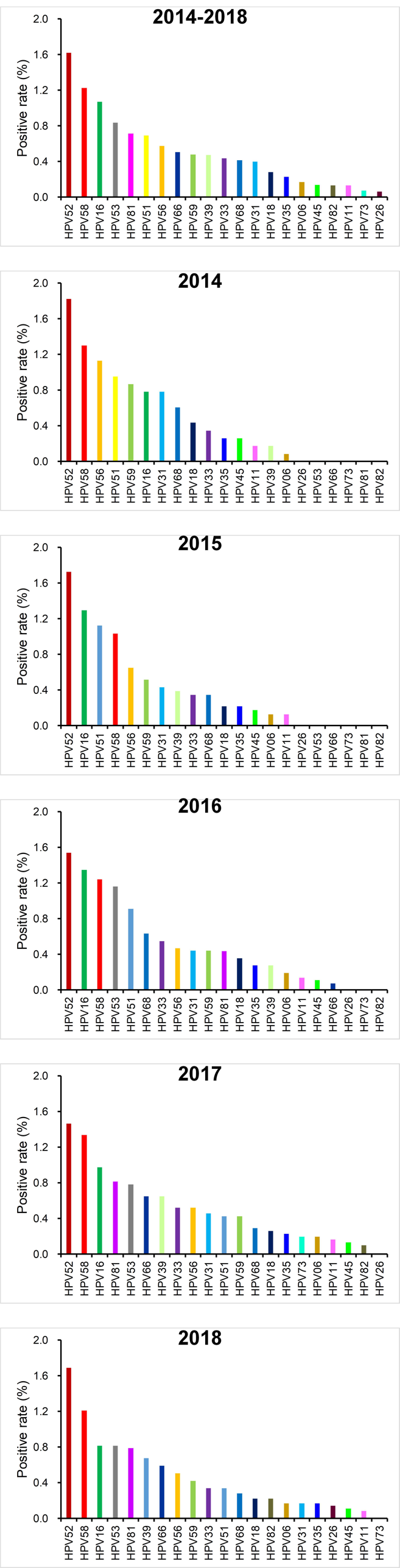

Supplement: Figure S3 [file peerj-08-8709-s003.png]

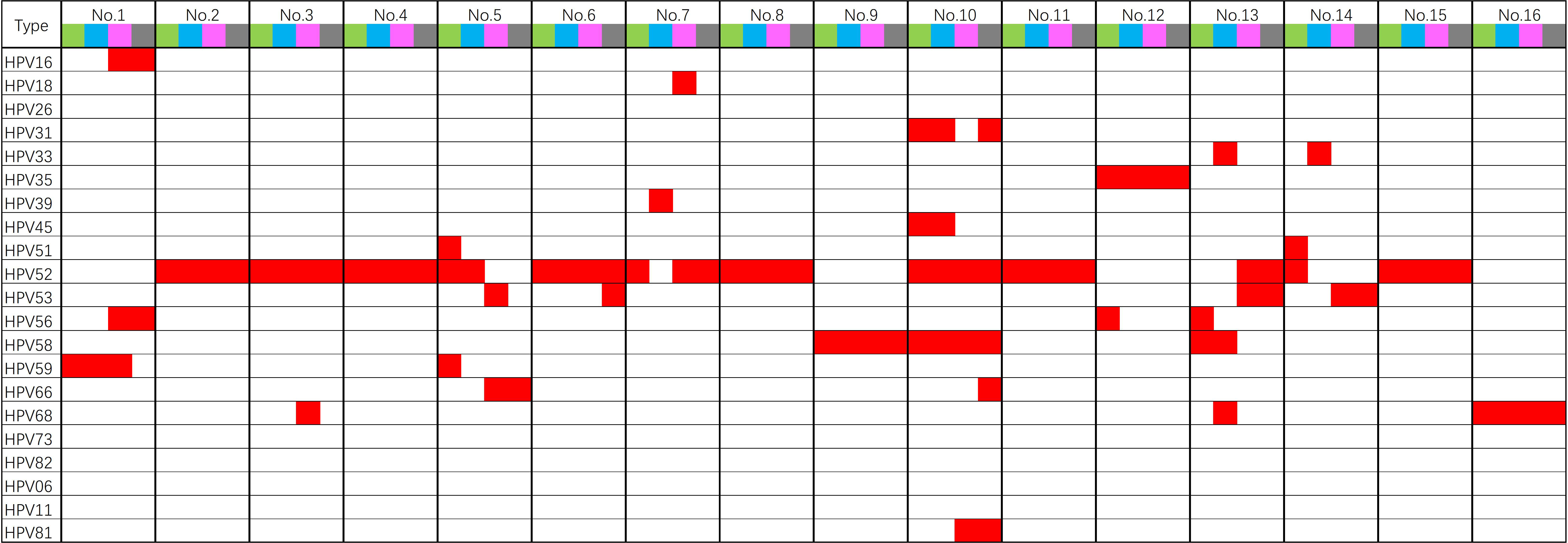

Supplement: Figure S4 [file peerj-08-8709-s004.png]
