# Supplementary material for: Prevalence and type distribution of human papillomavirus in a Chinese urban population between 2014 and 2018: a retrospective study
Source: PeerJ. 2020 Mar 23;8:e8709. doi: 10.7717/peerj.8709 (PMC7098390; doi:10.7717/peerj.8709)
Supplement: Table S1 [file peerj-08-8709-s005.docx]

Supplemental Table 1. The detailed information of two detection methods in the different years

| Variable | PCR reverse dot blot  (n=7703) | Fluorescence quantitative PCR  (n=6019) | P |
| --- | --- | --- | --- |
| Year, n |  |  | <0.001 |
| 2014 | 1050 | 103 |  |
| 2015 | 2152 | 166 |  |
| 2016 | 2252 | 1380 |  |
| 2017 | 0 | 3070 |  |
| 2018 | 0 | 3549 |  |
| Detection of HPV type, n (%) | |  |  |
| HPV06 | 13 (0.17) | 10 (0.17) | 0.970 |
| HPV11 | 12 (0.16) | 6 (0.10) | 0.368 |
