# Supplementary material for: Prevalence and type distribution of human papillomavirus in a Chinese urban population between 2014 and 2018: a retrospective study
Source: PeerJ. 2020 Mar 23;8:e8709. doi: 10.7717/peerj.8709 (PMC7098390; doi:10.7717/peerj.8709)
Supplement: Table S2 [file peerj-08-8709-s006.docx]

Supplemental Table 2. The distribution of two HPV types coinfection

| HPV type | 06 | 11 | 16 | 18 | 26 | 31 | 33 | 35 | 39 | 45 | 51 | 52 | 53 | 56 | 58 | 59 | 66 | 68 | 73 | 81 | 82 |
| --- | --- | --- | --- | --- | --- | --- | --- | --- | --- | --- | --- | --- | --- | --- | --- | --- | --- | --- | --- | --- | --- |
| **HPV06** | **13** | 1 | 1 | 0 | 0 | 0 | 1 | 0 | 1 | 0 | 2 | 0 | 1 | 2 | 3 | 0 | 1 | 1 | 0 | 0 | 0 |
| **HPV11** | 1 | **11** | 1 | 0 | 0 | 1 | 0 | 0 | 0 | 0 | 0 | 1 | 0 | 2 | 0 | 1 | 2 | 0 | 0 | 0 | 0 |
| HPV16 | 1 | 1 | **104** | 1 | 1 | 3 | 7 | 2 | 3 | 0 | 5 | 9 | 3 | 7 | 11 | 8 | 5 | 1 | 1 | 1 | 0 |
| HPV18 | 0 | 0 | 1 | **24** | 0 | 5 | 2 | 1 | 0 | 0 | 1 | 1 | 1 | 0 | 2 | 1 | 1 | 3 | 1 | 1 | 0 |
| HPV26 | 0 | 0 | 1 | 0 | **2** | 0 | 0 | 0 | 1 | 0 | 0 | 1 | 0 | 0 | 0 | 0 | 0 | 0 | 0 | 0 | 0 |
| HPV31 | 0 | 1 | 3 | 5 | 0 | **38** | 3 | 0 | 0 | 2 | 0 | 4 | 1 | 0 | 4 | 2 | 2 | 0 | 0 | 1 | 0 |
| HPV33 | 1 | 0 | 7 | 2 | 0 | 3 | **34** | 2 | 1 | 1 | 5 | 4 | 1 | 2 | 6 | 2 | 2 | 4 | 0 | 0 | 0 |
| HPV35 | 0 | 0 | 2 | 1 | 0 | 0 | 2 | **20** | 0 | 0 | 2 | 1 | 0 | 3 | 4 | 1 | 0 | 2 | 0 | 1 | 0 |
| HPV39 | 1 | 0 | 3 | 0 | 1 | 0 | 1 | 0 | **41** | 0 | 5 | 8 | 2 | 2 | 2 | 0 | 0 | 0 | 0 | 2 | 1 |
| HPV45 | 0 | 0 | 0 | 0 | 0 | 2 | 1 | 0 | 0 | **13** | 0 | 2 | 0 | 0 | 4 | 0 | 0 | 1 | 0 | 0 | 1 |
| HPV51 | 2 | 0 | 5 | 1 | 0 | 0 | 5 | 2 | 5 | 0 | **60** | 10 | 2 | 8 | 2 | 5 | 1 | 6 | 0 | 1 | 1 |
| HPV52 | 0 | 1 | 9 | 1 | 1 | 4 | 4 | 1 | 8 | 2 | 10 | **162** | 6 | 1 | 17 | 7 | 2 | 4 | 0 | 4 | 0 |
| HPV53 | 1 | 0 | 3 | 1 | 0 | 1 | 1 | 0 | 2 | 0 | 2 | 6 | **47** | 2 | 2 | 2 | 3 | 0 | 0 | 1 | 1 |
| HPV56 | 2 | 2 | 7 | 0 | 0 | 0 | 2 | 3 | 2 | 0 | 8 | 1 | 2 | **48** | 7 | 8 | 5 | 1 | 0 | 2 | 1 |
| HPV58 | 3 | 0 | 11 | 2 | 0 | 4 | 6 | 4 | 2 | 4 | 2 | 17 | 2 | 7 | **117** | 7 | 4 | 6 | 1 | 4 | 0 |
| HPV59 | 0 | 1 | 8 | 1 | 0 | 2 | 2 | 1 | 0 | 0 | 5 | 7 | 2 | 8 | 7 | **37** | 3 | 4 | 0 | 1 | 0 |
| HPV66 | 1 | 2 | 5 | 1 | 0 | 2 | 2 | 0 | 0 | 0 | 1 | 2 | 3 | 5 | 4 | 3 | **21** | 0 | 0 | 3 | 0 |
| HPV68 | 1 | 0 | 1 | 3 | 0 | 0 | 4 | 2 | 0 | 1 | 6 | 4 | 0 | 1 | 6 | 4 | 0 | **34** | 0 | 1 | 2 |
| HPV73 | 0 | 0 | 1 | 1 | 0 | 0 | 0 | 0 | 0 | 0 | 0 | 0 | 0 | 0 | 1 | 0 | 0 | 0 | **3** | 0 | 0 |
| **HPV81** | 0 | 0 | 1 | 1 | 0 | 1 | 0 | 1 | 2 | 0 | 1 | 4 | 1 | 2 | 4 | 1 | 3 | 1 | 0 | **44** | 0 |
| HPV82 | 0 | 0 | 0 | 0 | 0 | 0 | 0 | 0 | 1 | 1 | 1 | 0 | 1 | 1 | 0 | 0 | 0 | 2 | 0 | 0 | 6 |
